# The Efficient Antiviral Response of A549 Cells Is Enhanced When Mitochondrial Respiration Is Promoted

**DOI:** 10.3390/pathogens11101168

**Published:** 2022-10-11

**Authors:** Grégorie Lebeau, Daed El Safadi, Aurélie Paulo-Ramos, Mathilde Hoareau, Philippe Desprès, Pascale Krejbich-Trotot, Florian Chouchou, Marjolaine Roche, Wildriss Viranaicken

**Affiliations:** 1 Unité Mixte Processus Infectieux en Milieu Insulaire Tropical, Plateforme Technologique CYROI, Université de La Réunion, INSERM U1187, CNRS UMR 9192, IRD UMR 249, 94791 Sainte Clotilde, La Réunion, France; 2 INSERM, UMR 1188 Diabète Athérothombose Réunion Océan Indien (DéTROI), Université de La Réunion, 97400 Saint-Denis, La Réunion, France; 3 IRISSE Laboratory (EA4075), UFR SHE, University of La Réunion, 97430 Le Tampon, La Réunion, France

**Keywords:** antiviral response, ISGs, OXPHOS, mitochondrial respiration, metabolic reprogramming

## Abstract

When exposed to a viral infection, the attacked cells promptly set up defense mechanisms. As part of the antiviral responses, the innate immune interferon pathway and associated interferon-stimulated genes notably allow the production of proteins bearing antiviral activity. Numerous viruses are able to evade the interferon response, highlighting the importance of controlling this pathway to ensure their efficient replication. Several viruses are also known to manipulate the metabolism of infected cells to optimize the availability of amino acids, nucleotides, and lipids. They then benefit from a reprogramming of the metabolism that favors glycolysis instead of mitochondrial respiration. Given the increasingly discussed crosstalk between metabolism and innate immunity, we wondered whether this switch from glycolysis to mitochondrial respiration would be beneficial or deleterious for an efficient antiviral response. We used a cell-based model of metabolic reprogramming. Interestingly, we showed that increased mitochondrial respiration was associated with an enhanced interferon response following polyriboinosinic:polyribocytidylic acid (poly:IC) stimulation. This suggests that during viral infection, the metabolic reprogramming towards glycolysis is also part of the virus’ strategies to inhibit the antiviral response.

## 1. Introduction

The 21st century is a reminder of how humanity is constantly exposed to the danger of infectious diseases. Most of them are caused by the emergence or re-emergence of viral infectious agents. We had to face the severe acute respiratory syndrome coronavirus outbreak (in 2003), the swine flu pandemic (in 2009), the Middle East respiratory syndrome coronavirus outbreak (in 2012), the Ebola virus disease epidemic (between 2013 and 2016), the Zika virus disease epidemic (in 2015), and recently, the SARS-CoV-2 and the monkeypox outbreaks [1,2,3,4,5,6,7]. In addition to these particularly worrying pathogens, more neglected viruses such as Dengue virus or Chikungunya virus cause epidemics whose impact on populations should not be underrated [8,9]. In addition, developed countries are faced with an increase in the prevalence of metabolic disorders [10,11] and an aging population worldwide [12], especially in Western countries. These characteristics could correlate with the observed weakening of the population against severe forms of infectious diseases [13,14,15,16,17]. It also suggests the importance that the host metabolism may have on the outcome of viral infections. In line with this assumption, it has already been shown that many viruses are able to interact with the metabolism of their host to take advantage of it [18,19,20]. 

Physiologically, the metabolism of a quiescent cell relies on tricarboxylic acid cycle (TCA) and subsequent oxidative phosphorylation to produce an adequate amount of ATP, permitting to ensure all cellular functions [21]. Entry in TCA is mainly supported by pyruvate production, following intracellular glucose hydrolysis as the main source of tricarboxylic substrate [21]. Alternative tricarboxylic substrates exist and are, most of the time, derivatives of amino acids metabolism [21]. TCA allows the generation of reduced NADH and FADH_2_, which serve as electron transporters for the respiratory chain complexes [21]. Ultimately, oxidative phosphorylation, permitted by an aerobic environment and a suitable NADH and FADH_2_ generation, occurs at the inner membrane of mitochondria with conversion of potential energy to ATP [21]. Oxidative phosphorylation remains the most potent source of ATP since 36 ATP are produced for 1 molecule of glucose, whereas in non-aerobic conditions, 2 ATP are produced for 1 molecule of glucose [22]. Nevertheless, a metabolism based on oxidative phosphorylation is less efficient for the production of biomass (i.e., amino acids, nucleotides, and lipids), explaining why dividing cells perform aerobic glycolysis to ensure biomass availability [22]. In addition, as cells undergo anarchic divisions, cancer cells are known to redirect their metabolism towards aerobic glycolysis by a phenomenon called the Warburg effect [23]. Similarly, during infection, viruses have been shown to hijack host metabolism and direct it towards aerobic glycolysis in order to generate a sufficient pool of building blocks for their replication [19]. 

It is noteworthy that host metabolism was shown to be particularly crucial in mounting an effective immune response [24,25,26]. Indeed, a shift of metabolism towards glycolysis was observed in macrophages, which is necessary for M1 polarization and the establishment of its pro-inflammatory capabilities [27], while oxidative phosphorylation was associated with M2 polarization of macrophages and their anti-inflammatory and reparative functions [27]. Focusing on antiviral response and metabolism crosstalk, contradictory data were found depending on the virus studied: if some inhibit the mTOR pathway, others instead induce the AMPK pathway. Thus, the mTOR pathway was shown to be altered by different viruses via mTORC1 activation, including HSV1 [28], HIV-1 [29], and HCV [30]. mTOR pathway is known to regulate the expression of several glycolytic enzymes, thus promoting glycolysis. On the other hand, the AMPK pathway, which mediates mTORC1 inhibition, was shown to be both altered or promoted, respectively, during DENV infection and ZIKV infection [31,32]. Despite these conflicting data, it should be noted that glucose metabolism has an important place in the development of the antiviral response. Indeed, recently, a link between glucose metabolism and virus struggling has been established in vitro. Differential amounts of glucose in the culture medium of human kidney cells could lead to increased expression of antiviral effectors during ZIKV infection [33]. Conversely, oxidative phosphorylation could be a trigger for the development of an appropriate antiviral response. Supporting this hypothesis, it was shown that cells resistant to the induction of aerobic glycolysis promoted by HIV, which also perform more oxidative phosphorylation, had a higher antiviral potential accompanied by a control of the virus [20]. Reinforcing the link between cell respiration and viral infection, flaviviruses were shown to affect the respiratory pathway of infected cells by disrupting mitochondrial fission through the action of NS4B on mitofusins [34], thus preventing the initiation of a mitochondria-dependent antiviral state due to decreased ROS production [35].

During replication, numerous RNA viruses go through a stage where their genome is found as a double-stranded RNA (dsRNA) in the infected cell’s cytoplasm [36]. This dsRNA is recognized as a pathogen-associated molecular pattern (PAMP) by the pattern recognition receptors (PRRs) (i.e., endosomal Toll-like receptor 3 and cytosolic RIG-I-like receptors), leading to IRFs’ phosphorylation and translocation, ultimately resulting in type I IFNs’ (α/β) release [37,38,39]. In a paracrine and autocrine manner, the released IFNs will induce a signaling cascade via IFNs α/β receptors (IFNARs), leading to the production of antiviral proteins. The interferon-stimulated genes (ISGs) encoding antiviral proteins are under the control of promoters containing an ISRE or a gamma interferon activation site (GAS) element [40]. These antiviral effectors have various functions on the virus life cycle, including inhibition of viral entry, translation, replication, or even budding [41]. Alternatively, they are able to improve sensing or potentiate interferon signaling pathways [41]. The acute antiviral response is one of the first mechanisms to take place following infection, as it is the first line of defense preventing the virus from spreading across the organism [42]. Thus, this response is achieved by non-immune cells as part of innate immunity, pending the deployment of a specific adaptive immune response [42]. Notably, ISG56/IFIT1 and ISG54/IFIT2 are both members of the ISG56/IFIT1 gene family encoding P56 family proteins [43]. P56 proteins are cytoplasmic proteins with a tetratricopeptide repeat motif [43]. They do not possess enzymatic activity but are known to exert their antiviral properties in a non-specific manner through general inhibition of cell translation [43]. In parallel, more specific activities have been described against HCV, SINV, or HPV [43].

Herein, we wondered if increased OXPHOS could be beneficial for the development of antiviral response. To address this question, we set up a model of improved mitochondrial OXPHOS based on the work of Shiratori et al. [44]. Then, we studied the antiviral response through the expression and induction of antiviral genes (i.e., ISGs). Interestingly, we describe here an enhanced antiviral response in cells that mainly perform OXPHOS in contrast to cells whose metabolism depends mainly on glycolysis. This effect on antiviral response was shown to be based on mitochondrial function, as no improvement was observed when using rotenone, a mitochondrial OXPHOS inhibitor.

## 2. Materials and Methods

### 2.1. Cells and Reagents

The human A549-Dual™ (A549^Dual^) are adherent epithelial cells derived from A549 lung carcinoma cell line (ATCC, CCL-185™). A549D were purchased from InvivoGen (ref. a549d-nfis, San Diego, CA, USA). Lactate dehydrogenase (LDH) assay was performed using CytoTox 96^®^ Non-Radioactive Cytotoxicity assay from Promega (ref. G1780, Madison, WI, USA). Neutral red was purchased from Sigma-Aldrich (ref. N4638, Saint-Louis, MO, USA). Colorimetric lactate assay kit was purchased from Sigma-Aldrich (ref. MAK064, Saint-Louis, MO, USA). QUANTI-Luc™ substrate was obtained from InvivoGen (ref. rep-qlc1, San Diego, CA, USA). Rotenone was purchased from Sigma-Aldrich (ref. R8875, Saint-Louis, MO, USA). Poly(I:C) (HMW)/LyoVec™ (PIC) was purchased from InvivoGen (ref. tlrl-piclv, San Diego, CA, USA).

### 2.2. Cell Culture and Stimulation

A549^Dual^ cells were cultured for maintenance at 37 °C under a 5% CO_2_ atmosphere in 4.5 g·L^−1^ glucose Dulbecco’s Modified Eagle’s Medium (PAN Biotech, Aidenbach, Germany) supplemented with 10% of heat-inactivated fetal bovine serum (FBS, Dutscher, Brumath, France), 100 U·mL^−1^ penicillin, 100 μg·mL^−1^ streptomycin, 2 mM L-glutamine, and 0.5 μg·mL^−1^ amphotericin B (PAN Biotech, Dutsher, Brumath, France) and containing 10 µg·mL^−1^ blasticidin and 100 µg·mL^−1^ zeocin (InvivoGen, San Diego, CA, USA) for selection of cells expressing NF-κB-SEAP and IRF-Luc reporters.

Antiviral response was induced using polyriboinosinic:polyribocytidylic acid (poly:IC), a double-stranded RNA composed of an inosine strand and a cytidine strand, which was complexed with transfection reagents to improve its uptake. Poly:IC is used as a natural immunostimulant simulating viral infection. Based on Shiratori et al. [44], A549^Dual^ from passage 25 to 32 were cultured in RPMI-1640 (ref. P04-17550, PAN Biotech, Aidenbach, Germany) containing either glucose (2 g·L^−1^) or galactose (2 g·L^−1^) upon stimulation to promote metabolic reprogramming (Figure S1). Briefly, cells were plated in RPMI-1640 supplemented with 10% FBS containing 2 g·L^−1^ of glucose or 2 g·L^−1^ of galactose. After 24 h of adaptation, cells were stimulated during 24 h using PIC at 0.25 µg·mL^−1^. In the same manner, assessment of antiviral response following cellular respiration inhibition was achieved using PIC at 0.25 µg·mL^−1^ and rotenone at 1 µg·mL^−1^ during 24 h. Prior to these manipulations, we determined that the use of PIC at a concentration of 0.25 µg·mL^−1^ allowed the establishment of an effective antiviral response (Figure S3). In addition, the cytotoxicity of rotenone at 1 µg·mL^−1^ was evaluated (Figure S4). Following stimulation, cell culture supernatant was harvested and stored at –20 °C, while cells were lysed using RLT Lysis Buffer (Qiagen, Hilden, Germany) and stored at –80 °C for subsequent RNA extraction.

### 2.3. Cytotoxicity Assays

A549^Dual^ were plated in 96-well plate with a density of seeding corresponding to 10,000 cells per well. Cells were then treated, respectively, with DMEM, RPMI with glucose 2 g·L^−1^, or RPMI with galactose 2 g·L^−1^. DMEM–DMSO 10% was used as a positive death inductor. Then, 24 h after, the supernatant was harvested for LDH assay, and cells underwent neutral red assay.

LDH assay was achieved using CytoTox 96^®^ Non-Radioactive Cytotoxicity assay from Promega following manufacturer recommendations. Briefly, 50 µL of supernatant was mixed with 50 µL of LDH substrate and incubated at 37 °C for 15 min. Then, absorbance was read at 490 nm using a FLUOstar^®^ Omega (BMG LABTECH, Offenburg, Germany).

(1) $$Percent\;cytotoxicity=100\times \frac{Experimental\;LDH\;release\;\left(OD_{490}\right)}{Maximum\;LDH\;release\;\left(OD_{490}\right)}$$

Concomitantly, cell viability was assessed with neutral red uptake assay, as described by Repetto et al. [45]. Briefly, after removal of the cell culture supernatant, 100 µL of medium (DMEM, RPMI with glucose 2 g·L^−1^ or RPMI with galactose 2 g·L^−1^, respectively) containing 40 µg·L^−1^ of neutral red was added to each well. Cells were then incubated during 2 h at 37 °C. Following incubation, the medium was removed, and cells were washed with PBS. Finally, 150 µL of neutral red destain (50% ethanol, 49% H_2_O, 1% glacial acetic acid) was added to each well. Absorbance was read at 540 nm using FLUOstar^®^ Omega microplate reader (BMG LABTECH, Offenburg, Germany).

(2) $$Cell\;viability\;\left(\%\right)=100\times \frac{Experimental\;condition\;(OD_{540})}{Negative\;control\;(OD_{540})}$$

### 2.4. Phenol Red Absorbance Measurement

Phenol red was used to assess pH in cell culture medium [46]. Cells were cultured in RPMI with glucose 2 g·L^−1^ or RPMI with galactose 2 g·L^−1^. At cell confluence, culture medium was harvested, and pH was evaluated by measuring the absorbance at 450 and 560 nm. Absorbance spectra were obtained using Secomam UviLine 9600 (Aqualabo, Champigny-sur-Marne, France). Results were normalized to the absorbance of fresh medium.

### 2.5. Lactate Assay

Production of lactate was assessed in the cell culture supernatant using a colorimetric lactate assay kit (Sigma Aldrich, Saint-Louis, MO, USA), following manufacturer instructions. Briefly, cells were cultured in RPMI with glucose 2 g·L^−1^ or RPMI with galactose 2 g·L^−1^, and at confluence, cell culture supernatant was harvested. Then, samples were deproteinized and concentrated using a 10 kDa MWCO spin filter 10 min at 4000× *g*. Following this, 50 µL of supernatant was used for reaction and mixed with 50 µL of the master mix reaction. Absorbance was immediately read at 570 nm using Infinite^®^ 200 pro microplate reader (Tecan, Mannedorf, Switzerland). The amount of lactate present in each sample was determined from a lactate standard curve.

### 2.6. RT-qPCR

Total RNA was extracted from cell lysates by using the RNeasy Plus Mini Kit (cat. 74136, Qiagen, Hilden, Germany). The total cDNA was obtained by reverse transcription using random primers from Invitrogen (ref. 58875, ThermoFisher, Waltham, MA, USA) and M-MLV reverse transcriptase enzyme (ref. M1708, Promega, Madison, WI, USA) at 42 °C for 60 min. cDNA was then subjected to a quantitative polymerase chain reaction using a CFX96 Connect™ Real-Time Detection System (Bio-Rad, Hercule, CA, USA). For amplification, ABsolute™ Blue qPCR SYBR Green Low ROX mix (ref. AB-4322, ThermoFisher, Waltham, MA, USA) and specific primers were used to assess gene transcripts expression for following targets: ISG54 (F: 5′-CTGGTCACCTGGGGAAACTA-3′, R: 5′-GAGCCTTCTCAAAGCACACC-3′), ISG56 (F: 5′-GCAGCCAAGTTTTACCGAAG-3′, R: 5′-CACCTCAAATGTGGGCTTTT-3′), and RNA polymerase II (F: 5′-GCACCACGTCCAATGACAT-3′, R: 5′-GTGCGGCTGCTTCCATAA-3′). A threshold cycle (Ct) was calculated for each single sample amplification reaction in the exponential phase of amplification, using Bio-Rad CFX Manager 3.1 (Bio-Rad, Hercule, CA, USA). Data obtained were processed using the 2^–∆∆Ct^ method, with RNA polymerase II serving as the housekeeping gene.

### 2.7. Measurement of the IFN-β Pathway Activation

Activation of the interferon regulatory factors (IRF) pathway was monitored by measuring Lucia luciferase activity in A549^Dual^ cells. It was evaluated using QUANTI-Luc substrate according to manufacturer’s recommendations. Briefly, 100 µL of QUANTI-Luc substrate was added to 20 µL of cell culture supernatant, and IRF-induced luciferase levels were quantified by luminescence measurement using a Luminoskan™ microplate luminometer (ThermoFisher, Waltham, MA, USA). Results are presented as fold-change ratio of luminescence emitted by supernatant from treated cells in comparison with control cells.

### 2.8. Statistical Analyses

Statistical analyses were done by two-way ANOVA using Graph-Pad Prism software version 9. Values of *p* < 0.05 were considered statistically significant. Degrees of significance are indicated in the figure captions as follows: * *p* < 0.05; ** *p* < 0.001; *** *p* < 0.0002; **** *p* < 0.0001; ns = not significant.

## 3. Results

### 3.1. A549^Dual^ Is a Suitable Model for Metabolic Reprogramming Study

To assess the potential of mitochondrial OXPHOS on the efficiency of the antiviral response, we set up a metabolic reprogramming model based on a work recently published by Shiratori et al. [44]. In their work, Shiratori et al. showed that alternative culture methods for several cancer cell lines (PANC-1, A549, and HeLa) lead to a change in the metabolic pathways responsible for ATP production. The cell-growing conditions allow an upregulated mitochondrial function and OXPHOS instead of glycolysis for ATP production [44]. Thus, similarly to their methods, we cultured the epithelial cell line A549^Dual^ in RPMI-1640 supplemented with 10% FBS and only containing glucose 2 g·L^−1^ or galactose 2 g·L^−1^ (Figure S1).

**Figure 1:**
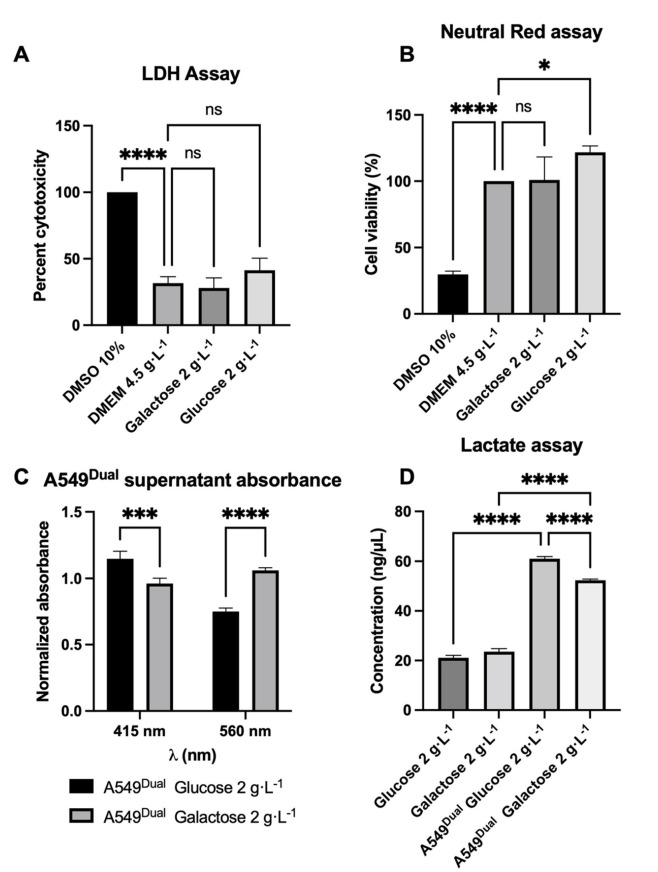
A549^Dual^ reprogramming from glycolysis to OXPHOS. (**A**,**B**) The different culture conditions do not affect A549^Dual^ viability. The RPMI medium supplemented with glucose 2 g·L^−1^ or galactose 2 g·L-1 does not affect cell viability as tested by both lactate dehydrogenase assay (**A**) and neutral red uptake assay (**B**) after 24 h of adaptation. DMEM-DMSO 10% was used for maximum death inducement. (**C**,**D**) The metabolism of cells grown in galactose 2 g·L^−1^ is less dependent on lactate production. Lactate production has been assessed in the supernatant of cells, first by evaluating the pH of cell culture supernatant (**C**) and then by using a commercial kit (**D**). Both experiments show a lactate production consistent with what was described by Shiratori et al. [44]; thus, A549^Dual^ cultured in galactose 2 g·L^−1^ have a metabolism shift from glycolysis to OXPHOS. For absorbance measurement, OD were normalized to OD of fresh culture medium. Error bars represent standard errors of three independent experiments. ns = not significant; * *p* < 0.05; *** *p* < 0.002; **** *p* < 0.0001.

We first had to determine if the specific culture conditions with glucose or galactose 2 g·L^−1^ would affect the viability of the A549^Dual^. To evaluate cell viability, we performed an LDH assay by measuring the activity of released LDH in the supernatant of our cells. As shown in Figure 1A, the cell culture conditions do not present any cytotoxicity. These results were confirmed by a neutral red uptake assay (Figure 1B).

It was previously described that cultivating cells in RPMI supplemented with galactose 2 g·L^−1^ increases OXPHOS and O_2_ consumption. This was notably evidenced, as ATP production was inhibited in the presence of oligomycin in contrast to cells cultured in glucose 2 g·L^−1^ [44]. Moreover, a decrease in lactate production due to the shift from glycolysis to OXPHOS characterized cells cultured in galactose [44]. In order to validate the identical behavior of our A549^Dual^ grown under the same conditions, we assessed the amount of lactate produced. To do so, we first measured the absorbance of the culture supernatant when cells reached confluence in the two culture conditions. The shift of phenol red from red to yellow is a potent indicator to assess the pH of the culture medium by measuring the absorbance at 415 nm and 560 nm [46]. Of note, it is known that diminution of pH in the culture medium is in part associated with production of lactate by the cells in culture [47]. Interestingly, we found that the supernatant of cells cultured in RPMI glucose 2 g·L^−1^ presents a higher absorbance at 415 nm and lower absorbance at 560 nm when compared to the cells cultured in RPMI galactose 2 g·L^−1^ (Figure 1C). For the same density of cells, this reflects acidification of the medium, which is more important for the cells cultured in glucose probably because of higher lactate production and release due to glycolysis.

To confirm this higher production of lactate and validate the suitability of our model for further experiments, we quantified the amount of lactate released under both culture conditions. Consistent with the previous result and with the work of Shiratori et al., we found that A549^Dual^ cultured in galactose 2 g·L^−1^ produce significantly less lactate than A549^Dual^ cultured in glucose 2 g·L^−1^ (Figure 1D). During all the experiments for the model characterization at the time of the analyses, the number of cells remains identical between the cells grown in glucose or galactose (Figure S2). The observed effects are not related to a difference in cell growth. Therefore, A549^Dual^ are a suitable cell model to study metabolic reprogramming and the impact of increased OXPHOS on the implementation of antiviral responses.

### 3.2. Metabolic Reprogramming Enhances Antiviral Response

Regular moderate physical activity appears to be associated with the development of less-severe disorders during infectious diseases [48,49,50]. In addition, a regular and moderate physical activity is associated with a higher mitochondrial activity and, consequently, an improved OXPHOS [51,52,53]. We wondered whether, at the cellular level, an increase in OXPHOS could be associated with the improvement of the antiviral response.

**Figure 2:**
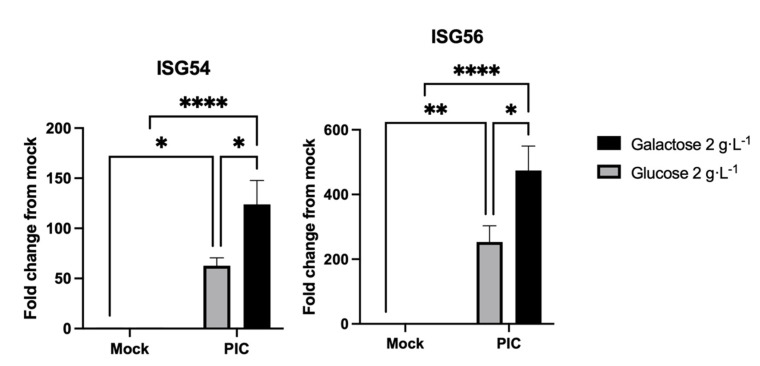
Metabolic reprogramming increases the expression of antiviral genes. ISG54 and 56 expressions have been assessed by RT-qPCR following a 24-h stimulation of A549^Dual^ either grown in glucose 2 g·L^−1^ or galactose 2 g·L^−1^ using PIC 0.25 µg·mL^−1^. Relative gene expression was obtained using RNA Polymerase II as a housekeeping gene. Fold-change results were obtained following normalization with relative gene expression of non-treated cells. Error bars represent standard errors of three independent experiments. * *p* < 0.05; ** *p* < 0.01; **** *p* < 0.0001.

To test this hypothesis, we stimulated A549^Dual^ with poly(I:C) (PIC), a dsRNA, which is widely used as a pathogen-associated molecular pattern mimetic. PIC is known to mimic replicating viral RNA and to trigger the canonical antiviral response, i.e., the IRF signaling pathway, through activation of TLR3-, RIG-I-, and RIG-I-like receptors (RLR), resulting in type I IFNs production and ISGs expression [54,55]. Thus, we assessed the expression of ISGs by A549^Dual^ in response to PIC stimulation. As presented in Figure 2, stimulation with PIC 0.25 µg·mL^−1^ induces a significant increase in ISG54 and ISG56 expression (at least 50- and 200-fold increase, respectively). Importantly, we report a greater ISGs expression inducement (up to a 2-fold difference) when the cells were grown in galactose 2 g·L^−1^ compared to the cells grown in glucose 2 g·L^−1^. This result indicates that cellular metabolism, depending on whether it is mainly glycolysis or OXPHOS pathway, has an effect on the antiviral response.

**Figure 3:**
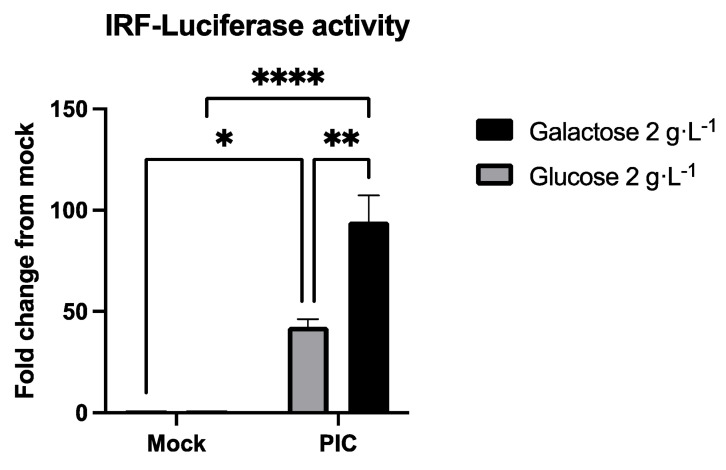
Metabolic reprogramming enhances the production of ISRE-dependent proteins. Analysis of IRF pathway activation in response to PIC 0.25 µg·mL^−1^ stimulation of A549^Dual^ either grown in glucose 2 g·L^−1^ or galactose 2 g·L^−1^. Activity of secreted Lucia luciferase was measured using QUANTI-Luc substrate 24 h after treatment. Results are expressed as a fold change of luminescence intensity between control cells and treated cells. Error bars represent standard errors of three independent experiments. * *p* < 0.05; ** *p* < 0.01; **** *p* < 0.0001.

To confirm the effect observed at the transcriptional level, we assessed the production of proteins dependent on the ISRE promoter. Indeed, A549^Dual^ are reporter cells Lucia luciferase under the control of an ISG54 minimal promoter in conjunction with five IFN-stimulated response elements (ISRE). Hence, we assessed the release of secreted luciferase in the supernatant of PIC-treated cells by measuring emitted luminescence using Lucia luciferase substrate (i.e., QUANTI-Luc). In agreement with RT-qPCR results, we observe a strong induction of secreted luciferase in response to PIC stimulation, representing a significant induction of antiviral proteins whose production is under the control of ISRE (Figure 3). Again, the induction is twice as high in cells grown in galactose 2 g·L^−1^ as in those grown in glucose 2 g·L^−1^ (Figure 3).

### 3.3. Antiviral Response Enhancement Relies on Cellular Respiration

From the precedent results, it is possible to argue that the differential induction of antiviral proteins in our two culture conditions relies on the improved OXPHOS of cells grown in galactose 2 g·L^−1^. However, to confirm this assumption, we decided to repeat the experiments in the presence of rotenone. Rotenone is a broad-spectrum insecticide, piscicide, and pesticide of vegetal origin, known to inhibit the complex I of mitochondrial electron transport chain [56], thus blocking OXPHOS and associated production of ATP [56].

**Figure 4:**
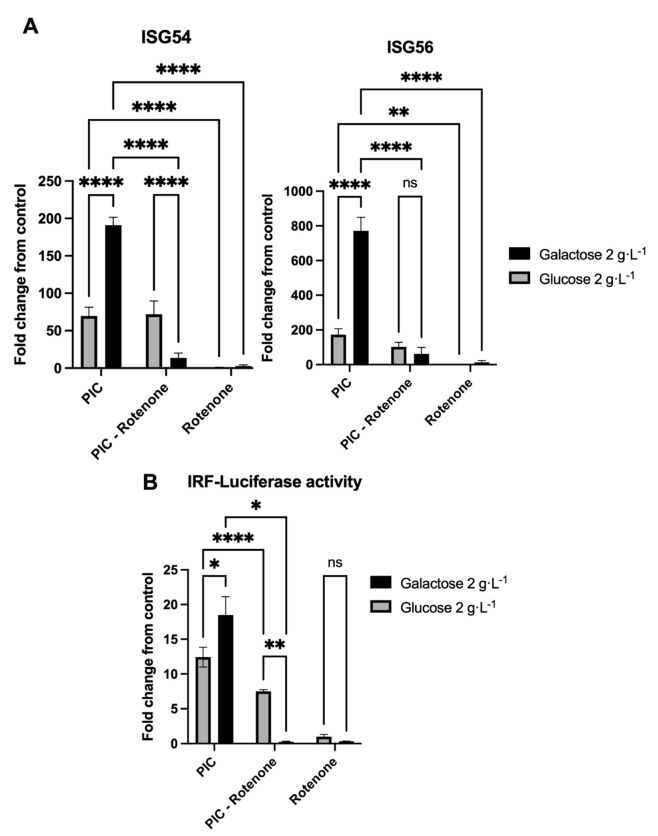
Antiviral enhancement relies on oxidative phosphorylation. (**A**) ISG54 and 56 expressions were assessed by RT-qPCR following a 24-h stimulation of A549^Dual^ either grown in glucose 2 g·L^−1^ or galactose 2 g·L^−1^ using PIC 0.25 µg·mL^−1^, rotenone 1 µg·mL^−1^, or both. Relative gene expression was obtained using RNA Polymerase II as a housekeeping gene. Fold change results were obtained following normalization with relative gene expression of non-treated cells. (**B**) Analysis of IRF pathway activation in A549^Dual^ either grown in glucose 2 g·L^−1^ or galactose 2 g·L^−1^ following stimulation with PIC 0.25 µg·mL^−1^, rotenone 1 µg·mL^−1^, or both. Activity of secreted Lucia luciferase was measured using QUANTI-Luc substrate 24 h after treatment. Results are expressed as a fold change of luminescence intensity between control cells and treated cells. Error bars represent standard errors of three independent experiments. Error bars represent standard errors of three independent experiments. ns = not significant; * *p* < 0.05; ** *p* < 0.01; **** *p* < 0.0001.

Following the addition of rotenone 1 µg·mL^−1^ ISGs expression was no longer observable in cells grown in galactose 2 g·L^−1^ and stimulated with PIC 0.25 µg·mL^−1^ compared to cells stimulated with PIC alone (Figure 4A). Furthermore, in comparison to cells grown in glucose 2 g·L^−1^, the expression of ISGs reached a lower level in the presence of rotenone, whereas no significant difference in expression was found for A549^Dual^ cultured in glucose and either treated or not with rotenone (Figure 4A). This result illustrates that the enhancement of ISGs expression in cells whose metabolism has been reprogrammed using galactose 2 g·L^−1^ essentially relies on improved OXPHOS. To further confirm this result, as previously, we assessed the induction of antiviral proteins using the IRF-luciferase reporter system permitted by A549^Dual^.

Remarkably, as shown in Figure 4B, similar results are obtained at the protein level, with luciferase gene expression and induction under the control of ISRE promoter. Indeed, a significant decrease is observed in antiviral protein production for the cells practicing OXPHOS and treated with both rotenone and PIC versus cells stimulated with PIC alone. As earlier, for cells grown in glucose 2 g·L^−1^ whose metabolism relies on glycolysis, the induction of antiviral proteins in presence of rotenone and PIC almost matches that of cells in presence of PIC alone (Figure 4B).

## 4. Discussion

Recently, the benefit of physical exercise on the development of severe forms of infectious diseases has been discussed, resulting in conflicting data [50,57,58]. Indeed, exercise demonstrated a protective effect against the development of severe forms of SARS-CoV-2 infection if the exercise was regular and moderate [48,49,59]. Of note, regular and moderate exercise is linked to the development of cellular respiration associated with a more effective mitochondrial oxidative phosphorylation [51,52,53]. On the other hand, intense or irregular sports practices are associated with a basal inflammatory state and lactate-dependent ATP production, which are favorable to viral infections [58]. Unfortunately, at the cellular level, the impact of mitochondrial oxidative phosphorylation on the establishment of a proper antiviral response has been poorly studied. However, Angin and colleagues reported increased antiviral potential against HIV in patient cells reprogrammed to boost their OXPHOS [20]. A study on this concern was necessary to emphasize the data obtained on patients, which mostly rely on retrospective studies.

To do so, we set up an in vitro model of improved mitochondrial oxidative phosphorylation in cultured cells, based on the work of Shiratori et al. [44]. Indeed, cancer cell lines are known to base their metabolism mostly on glycolysis and lactate production in order to optimize biomass production (e.g., nucleotides, amino acids, lipids) for more efficient cell proliferation [23]. Changing the growing medium from a glucose-containing medium to a galactose-containing medium redirects the cellular metabolism towards tricarboxylic acid cycle (TCA) and OXPHOS [44]. With galactose as a nutrient source, cells activate autophagic mechanisms leading to the release of glutamine, which, once converted into glutamate, serves as a substrate for TCA [44].

Using our validated model (Figure 1), we showed that cells with improved OXPHOS were able to mount an enhanced antiviral response through ISGs expression (Figure 2) and induction (Figure 3). Indeed, we reported an increased level of ISG54 and ISG56 expression in response to PIC stimulation. These genes, respectively, encode for IFIT2 and IFIT1 as antiviral effectors and are both under the control of two ISRE elements [43]. This justifies the use of the IRF–luciferase system permitted by A549^Dual^. Indeed, in these cells, the expression of Lucia luciferase is under the control of ISRE [60], reflecting the expression of ISRE-dependent ISGs.

Finally, to ensure that antiviral response enhancement was relying on mitochondrial respiration, we decided to stimulate A549^Dual^ this time with both PIC and rotenone, a complex I inhibitor widely used in mitochondrial function studies. As shown in Figure 4, ISGs were no longer expressed in cells grown in galactose 2 g·L^−1^ and treated with PIC and rotenone when compared to cells only treated with PIC. This indicates that potentiation of antiviral response described earlier relied on the improved mitochondrial function obtained in cells cultivated in galactose. Surprisingly, cells grown in glucose 2 g·L^−1^ had the same levels of ISG54 and ISG56 expression whenever treated with PIC or with both PIC and rotenone. This suggests that the glucose condition of cell culture leads to an antiviral response only dependent on glycolysis.

**Figure 5:**
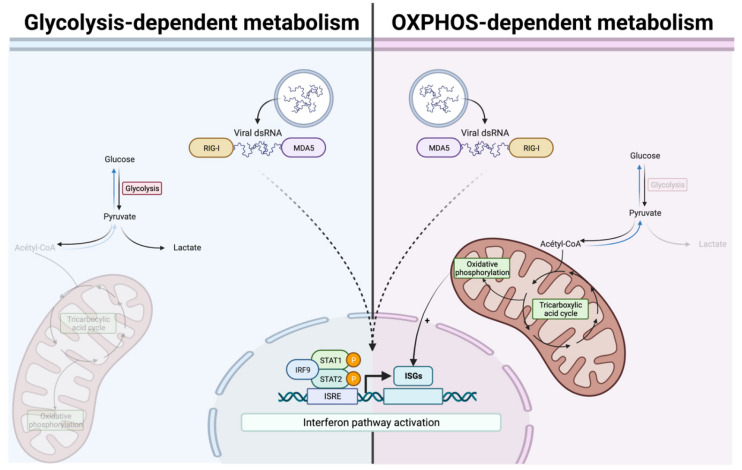
Enhancement of OXPHOS is associated with improvement of antiviral response.

Herein, we demonstrated an increased antiviral response in case of boosted OXPHOS (Figure 5). However, one limitation that can be argued is that we have demonstrated this for ISGs dependent on the ISRE element in their promoter. It would be interesting to further investigate whether the expression of ISGs with GAS elements in their promoter follows the same pattern. In addition, we would like to pursue this work by using primary cell cultures to overcome the Warburg effect of cancer cell lines [23]. This would allow us to be closer to what is observed physiologically under infection conditions, just adjusting the level of OXPHOS by using different amounts of glucose in the growth medium. On the other hand, it is known that during an infection, viral proteins often have an inhibitory activity on the antiviral response. This has been demonstrated for several viruses, such as the Dengue virus [61], the Zika virus [62], or even recently for the SARS-CoV-2 virus [63]. Thus, we attempted to overcome this inhibitory effect by using poly:IC, allowing us to have a general answer regarding the effect of OXPHOS on the development of the antiviral response. However, it remains important to determine whether the general mechanism we describe here is applicable to an infection with a replicating virus or whether more specific mechanisms are to be reported according to the family of viruses studied. To answer this, further studies are needed using several viruses, notably those of critical concerns nowadays.

## Data Availability

The data presented in this study are available in this paper.

## Supplementary Materials

The following supporting information can be downloaded at: https://www.mdpi.com/article/10.3390/pathogens11101168/s1.

## Abbreviations

**A549^Dual^**	A549-Dual™, expressing NF-κB-SEAP and IRF-Luc reporters
**GAS**	Gamma interferon activation site
**IFN**	Interferon
**IFNAR**	IFNs α/β receptor
**IRF**	Interferon regulatory factor
**ISG**	Interferon stimulated gene
**ISRE**	IFN-stimulated response element
**OXPHOS**	Oxidative phosphorylation
**PIC**	Poly(I:C) (HMW)/LyoVec™
