# Supplementary material for: The Efficient Antiviral Response of A549 Cells Is Enhanced When Mitochondrial Respiration Is Promoted
Source: Pathogens. 2022 Oct 11;11(10):1168. doi: 10.3390/pathogens11101168 (PMC9611969; doi:10.3390/pathogens11101168)
Supplement: Supplementary file 1 [file pathogens-11-01168-s001.zip › pathogens-1923503-supplementary.pdf]

pathogens

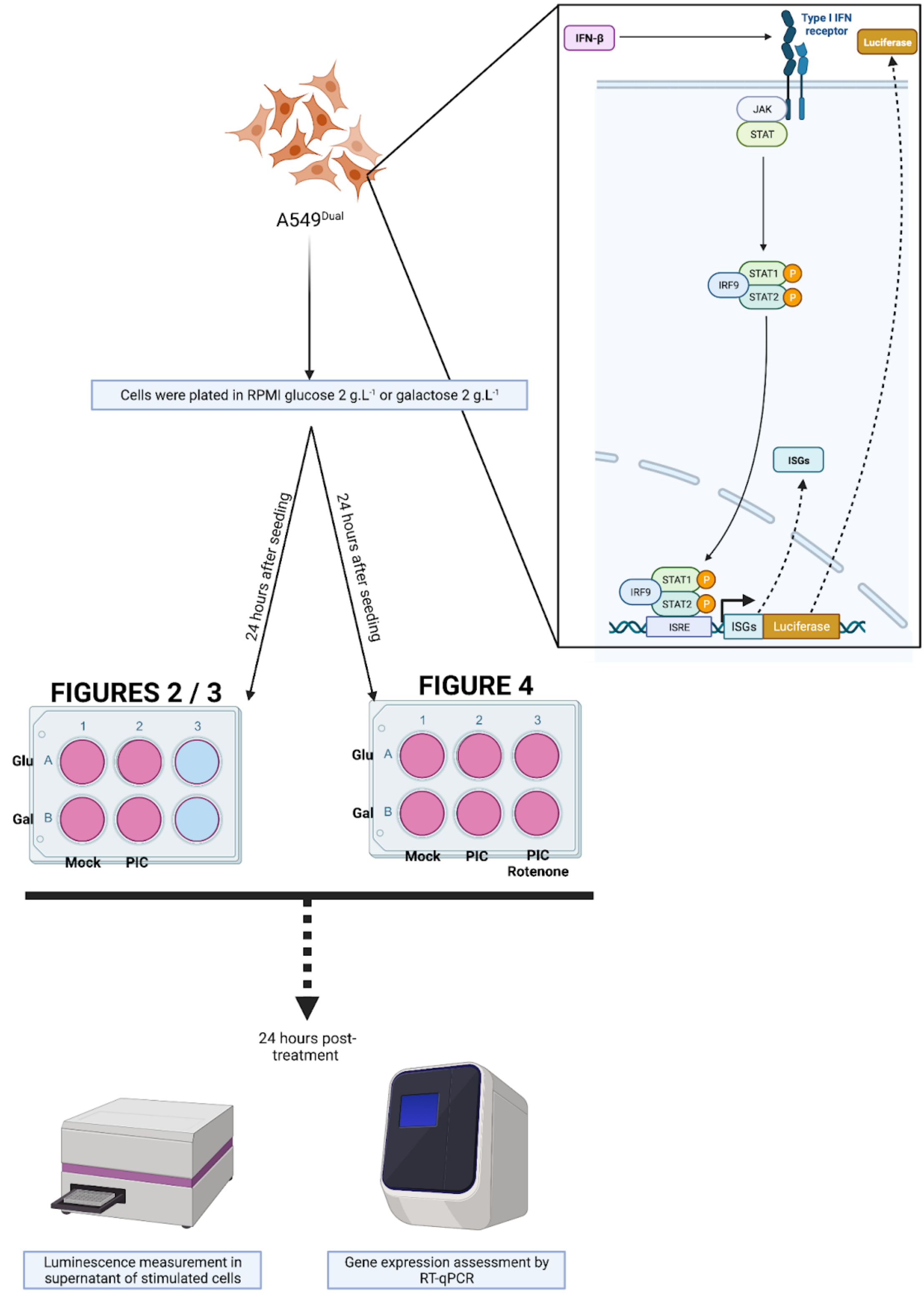

Figure S1. Experiments flowchart.

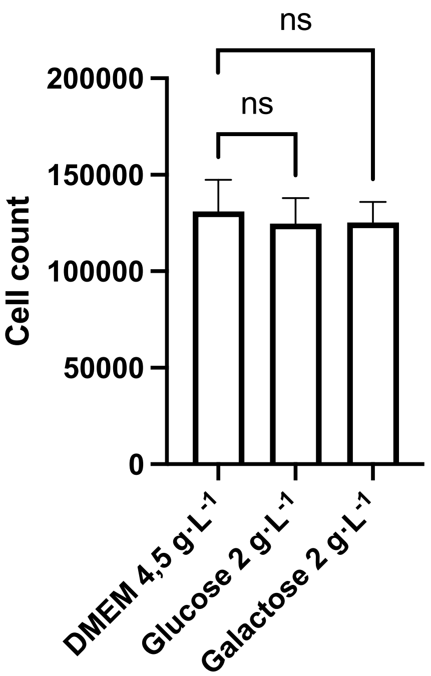

Figure S2. Cell growth comparison. A549^{Dual} were cultured in DMEM glucose 4,5 g·L^{-1} or RPMI glucose 2 g·L^{-1} / galactose 2 g·L^{-1}. Cells were seeded at the same density for each condition. Three days after seeding growth rate was assessed by counting cells using flow cytometry. This allowed us to determine that no significant difference in cell number was observable for the duration of the experiments. ns = not significant.

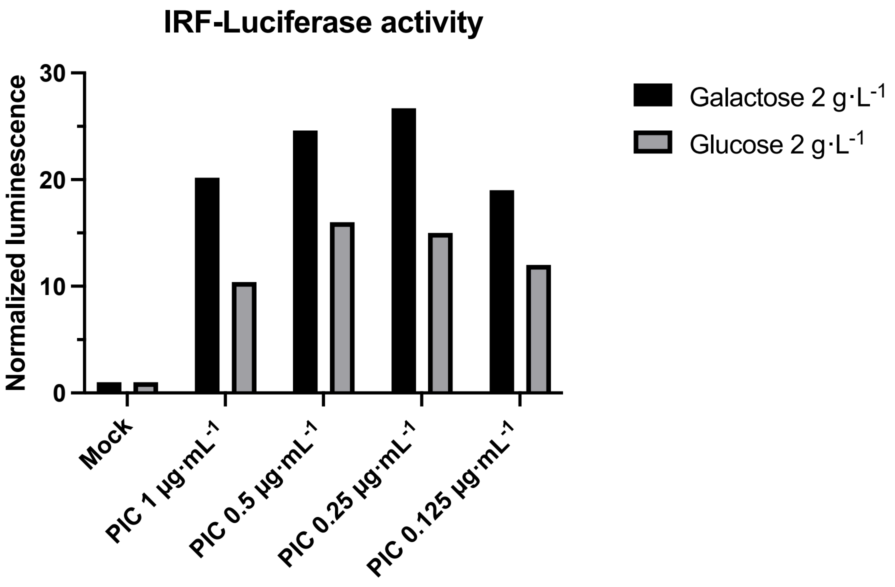

Figure S3. Poly:IC dose determination. We treated A549^{Dual} with poly:IC concentrations ranging from 1 µg·mL^{-1} to 0.125 µg·mL^{-1} for 24 hours. We then evaluated the impact of these different concentrations on establishment of antiviral response by measuring activity of secreted Lucia luciferase. Results are expressed as a fold change of luminescence intensity between control cells and treated cells.

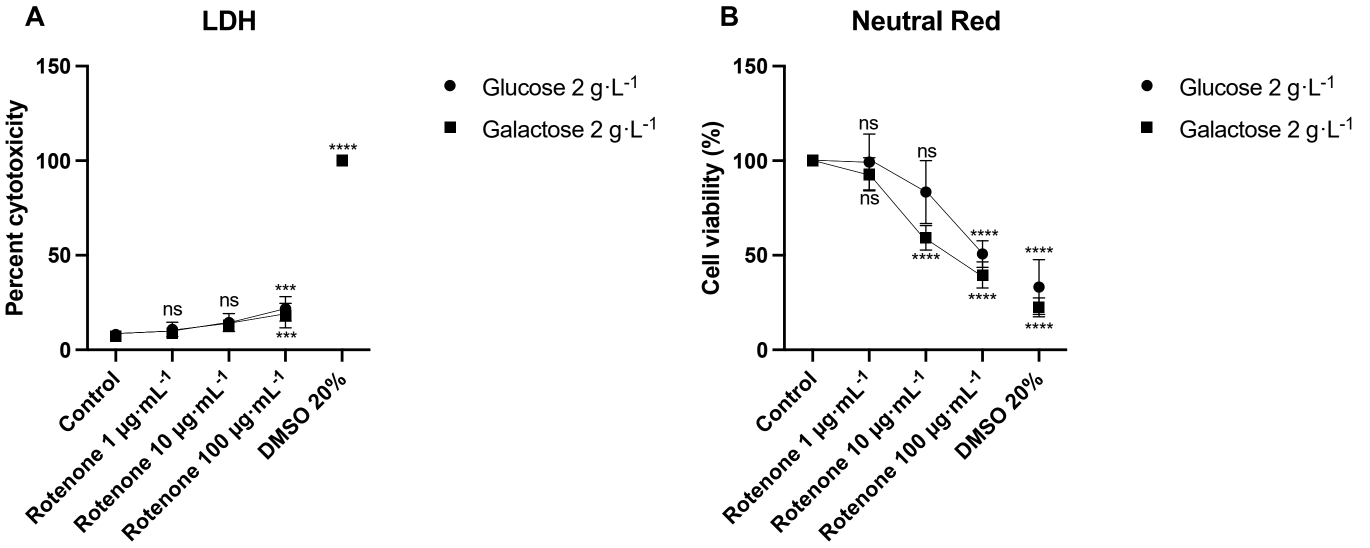

Figure S4. Cytotoxicity assessment of rotenone on A549^{Dual}. We treated A549^{Dual} with rotenone concentrations ranging from 1 µg·mL^{-1} to 100 µg·mL^{-1} for 24 hours. We then evaluated the impact of these different concentrations on cell viability by LDH assay and neutral red assay. ns = not sig-nificant; *** $p < 0.0002$; **** $p < 0.0001$.
